# Risk factors associated with cerebellar mutism syndrome and late postoperative hydrocephalus following pediatric posterior fossa medulloblastoma surgery

**DOI:** 10.3389/fonc.2026.1780096

**Published:** 2026-03-23

**Authors:** Jingxi Du, Fan Chen, Zhen Wang, Xiaoping Lv, Julei Wang, Tao Huang

**Affiliations:** 1 Department of Ultrasound Medicine, Tangdu Hospital of Air Force Medical University, Xi’an, China; 2 Department of Neurosurgery, Tangdu Hospital of Air Force Medical University, Xi’an, China; 3 Department of Medical Imaging, Hospital of Unit 96608, PLA, Hanzhong, China

**Keywords:** cerebellar mutism syndrome, children, late postoperation hydrocephalus, medulloblastoma, transvermian approach

## Abstract

**Objective:**

To investigate the risk factors for postoperative complications in pediatric patients with posterior cranial fossa medulloblastoma(MB).

**Methods:**

A retrospective analysis was performed based on the clinical data of 53 pediatric patients with posterior fossa medulloblastoma treated in Tangdu Hospital, from Dec, 2016 to May, 2024. Various data points, including age, gender, tumor maximum diameter, surgical approach, and complications, were collected and statistically evaluated for an association with cerebellar mutism syndrom(CMS) and late postoperation hydrocephalus (LPH).

**Results:**

The median age of 53 patients with MB was 6.75 years (interquartile range, 5.4–9.2years). The surgical approach used was the classic telovelar approach for 32 patients, the transvermian approach for 21 patients. Of the 53 children, 10 cases had developed cerebellar mutism syndrom and were included in the CMS group, with 43 patients in the non-CMS group. 9 cases had developed late postoperative hydrocephalus, Four patients were asymptomatic with normal cerebrospinal fluid (CSF) pressure on lumbar puncture and were managed conservatively with close clinical monitoring. the remaining five patients exhibited symptoms of elevated intracranial pressure—including headache and nausea—and demonstrated elevated CSF pressure on lumbar puncture; all underwent ventriculoperitoneal shunt placement. Statistical analysis revealed that tumor maximum diameter (OR 16.97, *P* = 0.011), transvermian approach (OR 97.02, *P* = 0.017) were independent risk factors for postoperative cerebellar mutism syndrome. Statistical analysis revealed that CSF-related complications (OR 212.7, *P* = 0.006), were independent risk factors for LPH.

**Conclusion:**

Our CMS rate is comparable to those reported in the literature. Despite the inherent limitations of the retrospective study design, our findings indicate that CMS was not only associated with the transvermian approach but also significantly linked to tumor’s maximum diameter. Furthermore, CSF-related complications were identified as independent risk factors for LPH.

## Introduction

CMS is a distinctive postoperative condition that typically manifests 1 to 2 days following the resection of a midline posterior fossa tumor. This syndrome is characterized by a reduction in speech that progresses to mutism, along with emotional lability, hypotonia, and ataxia (1). The delay in recognizing these symptoms may be due to the difficulties associated with conducting a comprehensive examination of a child during the immediate postoperative period. However, numerous studies have reported that CMS is generally not observed within the first few hours or days post-surgery (2–8). The onset of mutism and related neuropsychiatric symptoms following the resection of large vermian tumors was first documented by Hirsch et al (9). The trans-cerebellomedullary fissure approach (10–12), offers excellent exposure of the fourth ventricle without necessitating the splitting of the inferior vermis, thereby occasionally preventing the occurrence of the so-called postoperative vermian split syndrome (13–15). The prevailing understanding concerning the etiology of CMS points to damage inflicted upon the dentato-thalamo-cortical pathway (DTC pathway) (16).

Ahmadian et al. (17) conducted a systematic review and reported that cerebral perfusion is diminished in patients with CMS following posterior fossa tumor resection, compared to those without CMS. Similarly, Pettersson et al. (18) performed a systematic review to identify risk factors associated with the development of CMS. Their findings indicated that brainstem invasion, invasion of the fourth ventricle, invasion of the superior cerebellar peduncle, a diagnosis of MB, MB greater than 50 mm, left-handedness, and incision of the vermis are correlated with an increased incidence of CMS.

Drawing from the findings of this extant literature, we hypothesize that the tumor itself may not serve as the primary direct cause of CMS. Rather, the damage to or invasion of critical brain structures may underpin the development of CMS. Moreover, higher tumor burden—quantified by maximal diameter or volumetric measurement—is associated with an increased risk of brainstem adhesion and cerebellar vermis involvement (19), thereby elevating surgical complexity.

Findings from prior studies examining risk factors for posterior fossa syndrome remain inconsistent. Well-established risk factors include MB histology, vermian tumor location, and brainstem invasion (16, 20, 21), however, the association of tumor size (22, 23), preoperative hydrocephalus( (24), age at diagnosis (1, 25).and vermian (22) surgical approach with cerebellar mutism syndrome remains inconclusive in the current literature. Consequently, we conducted a focused investigation into factors associated with tumor size, surgical variables—encompassing the surgical approach and incision of the cerebellar vermis—and patient-specific characteristics, to identify potential risk factors for CMS.

This study aims to systematically evaluate surgical outcomes in pediatric posterior fossa medulloblastoma and to identify independent risk factors associated with postoperative complications, with particular emphasis on modifiable factors amenable to preoperative optimization or perioperative interventions for the prevention or mitigation of complications.

## Methods

### Patients

We conducted a retrospective, single-center study, which received approval from our institutional ethics committee for data collection. Written informed consent was obtained from the parents or guardians prior to data collection. A retrospective analysis was performed on the clinical data of 53 pediatric patients diagnosed with posterior fossa medulloblastoma and treated at the Department of Neurosurgery, Tangdu Hospital, Air Force Medical University, between Dec, 2016 and May, 2024.Data were retrospectively extracted from medical records and included the following variables: gender, weight, height, symptoms associated with trunk ataxia prior to surgery, postoperative clinical course, presence of hydrocephalus (both preoperative and postoperative), maximum tumor diameter, histopathological findings, CSF-related complications (such as CSF leak, pseudomeningocele and meningitis), and the surgical approach. The maximum tumor diameter was determined by reviewing axial MRI studies and measuring the largest transverse diameter.

The inclusion criteria were primary posterior fossa medulloblastoma, age ≤ 18 years. This study involved patients who were receiving surgical treatment for the first time in our center.

Exclusion criteria encompassed cases of tumor metastasis, Patients undergoing non-primary surgical procedures, the child demonstrated no verbal abilities preoperatively and those who experienced cerebellar infarction.

### Therapeutic method

All patients underwent resection of the tumor via the suboccipital posterior midline approach. Incision length was tailored to tumor volume, and resection of the posterior arch of the atlas was performed selectively based on intraoperative anatomical requirements. A nuchal ligament fascial flap measuring approximately 3 cm × 3 cm was harvested intraoperatively and used for dural reconstruction. Adjuvant radiotherapy combined with chemotherapy was initiated postoperatively.

### Statistics

The IBM SPSS 21.0 (IBM Corp., NY, USA) was used for data analysis. Normal distribution of data was analyzed by Kolmogorov-Smirnov test and was expressed as the mean ± SD. Non-normally distributed data and ordinal data were expressed as median with inter-quartile range (IQR). Unpaired *t*-test was used for the comparisons of parameters with normal distribution between two groups and Mann-Whitney U test was used when in parameters without normal distribution. Categorical data was expressed as number (percentage) and compared by Chi-square χ² test or Fisher’s exact test. A two-sided *P* value < 0.05 was regarded as statistically significant.

## Results

In this study, we analyzed a cohort of 53 pediatric patients who underwent resection of posterior fossa medulloblastoma. The median age of the participants was 6.75 years, with an interquartile range of 5.40 to 9.20 years. The surgical techniques utilized included the classic telovelar approach in 32 patients and the transvermian approach in 21 patients. Postoperative complications related to cerebrospinal fluid were observed in 9 cases, which included 3 instances of CSF leak, 3 instances of pseudomeningocele, and 3 instances of meningitis. Among the 53 children, 10 experienced postoperative CMS, while 9 patients developed long-term postoperative hydrocephalus. We conducted a comparative analysis of surgical parameters and complications between the CMS group and the non-CMS group in pediatric posterior fossa medulloblastoma cases. The study identified that cerebellar mutism syndrome occurred in two cases within the telovelar approach group, compared to eight cases in the transvermian approach group, with this difference being statistically significant (*P* = 0.009). Additionally, the maximum tumor diameter demonstrated statistical significance (*P* = 0.003), and the length of hospital stay was significantly reduced (*P* = 0.012), as detailed in **Table 1**.

**Table 1:** Analysis of relevant factors for postoperative cerebellar mutism.

Variable	Overall, N = 53^1^	CMS N = 10 (19%)^1^	Non-CMS N = 43 (81%)^1^	Statistics	*P*-value^2^
Age (years)	6.70 [5.40, 9.20]	5.50 [4.65, 7.28]	6.70 [5.60, 9.60]	156.00	0.183
Gender					>0.999
Male	29 (54.72%)	6 (60.00%)	23 (53.49%)		
Female	24 (45.28%)	4 (40.00%)	20 (46.51%)		
Tumor Maximum Diameter (cm)	3.70 [3.10, 5.10]	5.10 [4.65, 5.20]	3.50 [3.10, 3.90]	347.00	0.003
Preoperative Trunk Ataxia				0.45	0.503
Yes	19 (35.85%)	5 (50.00%)	14 (32.56%)		
No	34 (64.15%)	5 (50.00%)	29 (67.44%)		
Hospital LOS (days)	9.00 [7.00, 10.00]	11.00 [9.25, 16.75]	9.00 [7.00, 10.00]	324.00	0.012
Preoperative EVD					0.667
Yes	11 (20.75%)	1 (10.00%)	10 (23.26%)		
No	42 (79.25%)	9 (90.00%)	33 (76.74%)		
Surgical Approach					0.009
Transvermian	21 (39.62%)	8 (80.00%)	13 (30.23%)		
Telovelar	32 (60.38%)	2 (20.00%)	30 (69.77%)		
Late Postoperative Hydrocephalus					0.346
Yes	9 (16.98%)	3 (30.00%)	6 (13.95%)		
No	44 (83.02%)	7 (70.00%)	37 (86.05%)		
CSF-related Complications					
CSF leak					0.088
Yes	3 (5.66%)	2 (20.00%)	1 (2.33%)		
No	50 (94.34%)	8 (80.00%)	42 (97.67%)		
Pseudomeningocele					>0.999
Yes	3 (5.66%)	0 (0.00%)	3 (6.98%)		
No	50 (94.34%)	10 (100.00%)	40 (93.02%)		
Meningitis					>0.999
Yes	3 (5.66%)	0 (0.00%)	3 (6.98%)		
No	50 (94.34%)	10 (100.00%)	40 (93.02%)		

^1^Median [IQR]; n (%).

^2^Wilcoxon rank sum test; Fisher’s exact test; Pearson’s Chi-squared test.

CMS, cerebellar mutism syndrome; LOS, length of stay; EVD, external ventricular drain.

**Figure 1:**
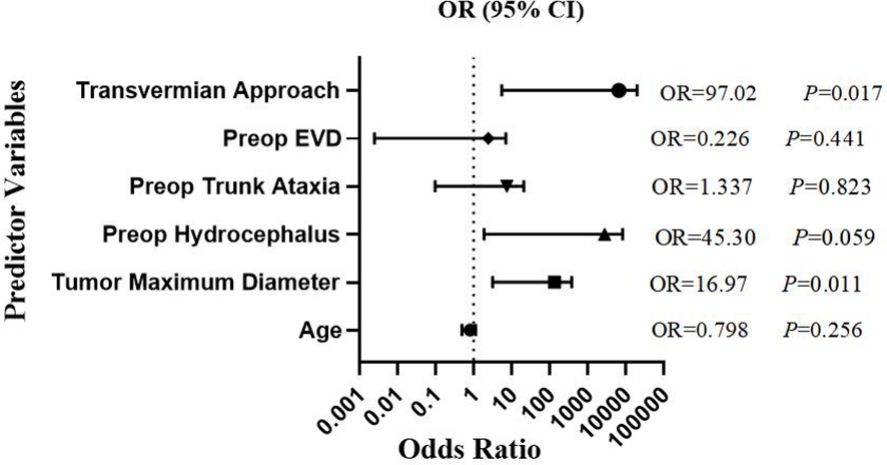
Forest plot showing odds ratio of multivariable logistic regression for occurrence of mutism. Risk variable indicated in brackets (Preop, preoperative; EVD, external ventricular drain).

Univariate and multivariate logistic regression were performed to explore risk factors for postoperative complications (**Table 2**). Variables with *P* < 0.5 in the univariate logistic regression analysis (age, gender, tumor maximum diameter, pathological type, preoperative hydrocephalus, preoperative trunk ataxia, preoperative EVD, transvermian approach) were included in the multivariate logistic regression analysis. Statistical analysis revealed that tumor maximum diameter (OR 16.97, *P* = 0.011), transvermian approach (OR 97.02, *P* = 0.017) were independent risk factors for postoperative cerebellar mutism syndrome. Forest plot (**Figure 1**) were utilized to analyze the association between cerebellar mutism and six additional contributing factors(age, tumor maximum diameter, preop hydrocephalus, preop trunk ataxia, preop EVD, transvermian approach).

**Table 2:** Univariate and multivariate regression analysis of risk factors for cerebellar mutism syndrome.

Variable	Univariate Analysis		Multivariate Analysis	
	OR (95% CI)	*P* Value	OR (95% CI)	*P* Value
Age	0.894 (0.662 to 1.146)	0.411	0.798 (0.495 to 1.132)	0.256
Gender	0.767 (0.175 to 3.071)	0.710		
Tumor Maximum Diameter	4.505 (1.873 to 13.55)	0.002	16.97 (3.175 to 384.0)	0.011
Pathological Type	0.800 (0.210 to 2.150)	0.691		
Preop Hydrocephalus	1.931 (0.415 to 13.92)	0.442	45.30 (1.881 to 8435)	0.059
Preop Trunk Ataxia	2.071 (0.501 to 8.637)	0.306	1.337 (0.098 to 21.12)	0.823
Preop EVD	0.367 (0.019 to 2.325)	0.368	0.226 (0.003 to 7.122)	0.441
Transvermian Approach	9.231 (1.991 to 67.02)	0.010	97.02 (5.563 to 20016)	0.017

Preop, preoperative.

**Figure 2:**
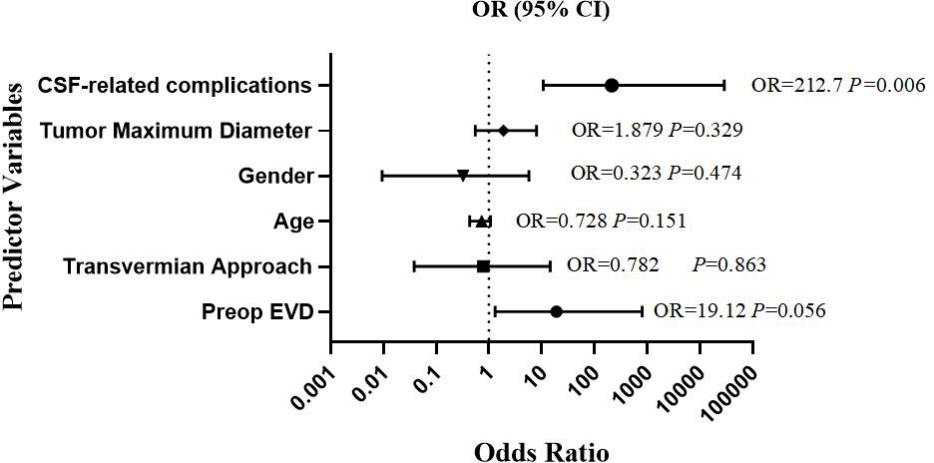
Forest plot showing odds ratio of multivariable logistic regression for occurrence of late postoperative hydrocephalus. Risk variable indicated in brackets(Preop, preoperative; EVD, external ventricular drain).

Univariate and multivariate logistic regression were performed to explore risk factors for postoperative hydrocephalus exacerbation (**Table 3**). Statistical analysis revealed that CSF-related complications (OR 212.7, *P* = 0.006), were independent risk factors for postoperation hydrocephalus exacerbation. Forest plot (**Figure 2**) were employed to analyze the association between postoperative hydrocephalus exacerbation and six contributing factors: age, tumor maximum diameter, gender, preop EVD, transvermian approach, cerebrospinal fluid (CSF)-related complications.

**Table 3:** Univariate and multivariate regression analysis of risk factors for late postoperative hydrocephalus.

Variable	Univariate Analysis		Multivariate Analysis	
	OR (95% CI)	*P* Value	OR (95% CI)	*P* Value
Age	0.830 (0.582 to 1.093)	0.233	0.7284 (0.431 to 1.072)	0.151
Gender	2.889 (0.670 to 15.13)	0.169	0.3228 (0.009 to 5.738)	0.474
Tumor Maximum Diameter	1.012 (0.435 to 2.229)	0.976	1.879 (0.552 to 7.972)	0.329
CSF-related complications	27.33 (4.951 to 202.2)	0.0004	212.7 (10.74 to 29045)	0.006
Preop EVD	4.229 (0.867 to 20.36)	0.067	19.12 (1.309 to 801.1)	0.056
Transvermian Approach	1.271 (0.280 to 5.468)	0.746	0.782 (0.0382 to 14.49)	0.863

Preop, preoperative; CSF-related complications (CSF leak, Pseudomeningocele, Meningitis).

## Discussion

MB is the most common malignant brain tumor in children. MB is a highly malignant embryonal tumor or primitive neuroectodermal tumor of the cerebellum with propensity for leptomeningeal dissemination. It is classified as a World Health Organization (WHO) grade 4 tumor. In 1910, Dr. James Wright firstly described a tumor in the cerebellum that resembled neuroblastoma and named it as “neurocytoma” (26). The terminology “medulloblastoma” was firstly introduced in 1925 by Bailey and Cushing, who characterized the tumor as a highly malignant glioma originating in the fourth ventricle, with a noted propensity for dissemination throughout the central nervous system (27), The management of medulloblastoma encompasses surgical intervention, chemotherapy, and irradiation. The significance of surgery and irradiation has been acknowledged since Cushing and Bailey’s initial report in 1925 (27). The primary site of treatment failure in these patients was identified as the leptomeninges within the brain and spinal cord. In a 1952 study by Cuneo and Rand involving 22 cases of medulloblastoma, 13 patients underwent radiotherapy, with survival durations ranging from 4 to 30 months, compared to 5 days to 4 months for those not receiving irradiation. Notably, none of these patients achieved a gross total resection of their primary tumor (8). In children older than 3 years of age, the long-term survival can be achieved in approximately 85% of standard risk patients and 70% of high risk patients with a combination of chemotherapy and irradiation (28), Despite tremendous progress in the field of molecular biology of medulloblastoma, much remains to be achieved in understanding the complications, For example, cerebellar mutism syndrome, cerebrospinal fluid leakage, and in devising treatment strategies with even better survival and less long-term sequelae.

Postoperative cerebellar mutism syndrome (pCMS), also interchangeably referred to as posterior fossa syndrome (PFS) (29), is a severe neurological complication following resection of a posterior fossa tumor (30). Despite pCMS having a low incidence in adults (31), children are the most susceptible to the syndrome, with a reported risk ranging from 8% to 39% (1), therefore, identifying the risk factors for cerebellar mutism syndrome, minimizing postoperative complications, and timely initiating radiotherapy and chemotherapy are essential to further improve patient outcomes.

Cerebellar mutism, a temporary issue with an uncertain anatomical basis, typically occurs following the removal of midline tumors affecting the vermis, Mutism following posterior fossa surgery was first reported more than 40 years ago (9). Elaborations on these early descriptions were subsequently noted in reports of more than 200 cases that had been retrospectively ascertained, These case reports have contributed to a deeper understanding of what has come to be known as cerebellar mutism syndrome, or posterior fossa syndrome (32–34), Cerebellar mutism is a significant and potentially underrecognized complication affecting many children who have undergone posterior fossa surgery for tumor removal, particularly when the tumor is situated in the midline. This research contributes to the current evidence linking damage to the superior cerebellar peduncle with cerebellar mutism syndrome following medulloblastoma removal (35).

The clinical manifestations of pCMS predominantly include postoperative delayed mutism, dysarthria, altered speech rate, grammatical disturbances, diminished attention and memory, reduced spontaneous movements, hypotonia, ataxia, urinary retention, dysphagia, personality changes, apathy, irritability, and autism. Notably, the mutism is generally transient, typically resolving within several months to six months postoperatively, with dysarthria frequently emerging during the recovery phase. Additionally, mutism often exhibits a latency period, usually manifesting within one week post-surgery (16). However, exceptions to this pattern exist. For instance, Korah et al (25), documented six pediatric cases in which mutism developed immediately following surgery. Some children displayed isolated impairments in language function, specifically manifesting as inaccurate pronunciation, dysarthria, and a slowed speech rate. During the acute phase, the majority of affected children experienced progressively worsening mutism, transitioning fromxreduced speech to complete muteness, often accompanied by emotional instability and ataxia (36), with dysarthria potentially emerging during the recovery period following mutism resolution (20).

The sequence of symptom recovery in pCMS is as follows: psychiatric symptoms, motor dysfunction, urinary retention, swallowing function, and speech function. However, cognitive and psychological impairments, motor dysfunction, and ataxia often persist despite the resolution of other symptoms (37). Andrew et al. (38) suggested that Current concepts in cerebellar physiology highlight the critical role of the cerebellum in learning and language. The syndrome described is indicative of a loss of learned activities, specifically apraxia, which affects the oral and pharyngeal musculature. To mitigate the risk of developing apraxia, it is crucial to preserve the inferior vermis. For large midline tumors extending to the aqueduct, a combined surgical approach via the fourth ventricle with a midvermis split can be utilized to minimize the risk of damage to the inferior vermis. In summary, pCMS is a clinical syndrome characterized by impairments in speech, motor function, cognition, and emotion. Conducting a thorough analysis of the onset sequence and duration of these symptoms is critically important for elucidating the interrelationships among them and achieving a more comprehensive understanding of this syndrome.

MB originates from the cerebellar vermis and extends into the fourth ventricle. If the tumor can be completely resected along the natural cerebello-medullary fissure, minimal injury is anticipated, potentially leading to favorable outcomes. It is crucial to emphasize that the telovelar approach should be adapted flexibly according to specific clinical circumstances. When a tumor demonstrates a highly vascularized nature, as observed in the present case, is substantial in size, adheres firmly to the brainstem, and exhibits a hard and resilient texture, it presents considerable challenges in achieving adequate exposure through the restricted medullary fissure. Specifically, the tasks of severing the tumor’s blood supply and delineating the interface between the tumor and the brainstem are particularly arduous and require exceptional surgical expertise. Consequently, a multimodal approach is recommended to facilitate the successful resection of the tumor.

San Y. C. V. Pols et al. (39) and Pettersson et al. (18) proposed that MB with maximum diameter represent significant risk factors for the development of CMS. Our study corroborates this finding, aligning with the result reported in the aforementioned research. Increased tumor volume substantially elevates surgical complexity: First, preoperative involvement of peritumoral normal brain parenchyma expands, and the brainstem frequently exhibits dense adhesions or frank invasive infiltration. Second, mechanical compression and displacement of adjacent neurovascular structures become more pronounced, necessitating wider surgical exposure, greater retraction, and consequently larger operative corridors and dural/brain surface incisions. Collectively, these anatomical and technical challenges heighten the risk of inadvertent bilateral dentate nucleus injury intraoperatively, thereby contributing to a higher incidence of CMS. The results of our research are consistent with those reported by Grønbæk et al (40). A key limitation of this study is the heterogeneity in surgical strategy for giant MB including variations in surgical approach, sequencing of resection steps, intraoperative neuromonitoring protocols, and extent of decompressive maneuvers—across institutions. Such procedural variability may introduce confounding bias in the assessment of CMS incidence and other critical neurocognitive outcomes.

### Study limitations

The sample size included in this study was relatively small, which led to the results being somewhat biased. It is important to note that refinements in surgical protocols, advancements in medical instrumentation, and enhancements in surgeons’ expertise, among other complex factors, may introduce potential biases. Therefore, we exercised considerable caution in interpreting the results and drawing conclusions. Due to the retrospective nature of our study, our data were dependent on clinical documentation, which may have been incomplete. Although handedness has been previously identified as a potential risk factor for CMS, it was not adequately documented within our cohort. Additionally, mild cases of cerebellar mutism syndrome (CMS) may have been underreported.

## Conclusions

Within the constraints of a retrospective, single-institutional study, we have detailed our institutional experience with cerebellar mutism syndrome (CMS) following posterior fossa tumor surgery in a pediatric cohort. This study identifies maximum tumor diameter and the transvermian surgical approach as independent risk factors for cerebellar mutism syndrome, and statistical analyses indicated that complications related to cerebrospinal fluid (CSF) were independent risk factors for the late postoperative hydrocephalus.

## Data Availability

The original contributions presented in the study are included in the article/supplementary material. Further inquiries can be directed to the corresponding authors.
